# Adaptive Judo and neuropathy: a mini review on motor skills, balance, and quality of life improvement

**DOI:** 10.3389/fpsyg.2024.1545358

**Published:** 2025-02-04

**Authors:** Erika Nerozzi, Valeria Prada, Francesco Pegreffi, Marina Grandis, Angelo Schenone, Emanuela Pierantozzi

**Affiliations:** ^1^Department for Life Quality Studies, University of Bologna, Bologna, Italy; ^2^Fondazione Italiana Sclerosi Multipla, FISM, Genoa, Italy; ^3^Department of Neuroscience, Rehabilitation, Ophthalmology, Genetics, and Mother-Child, School of Medical and Pharmaceutical Sciences, University of Genoa, Genoa, Italy; ^4^Department of Medicine and Surgery, School of Medicine and Surgery, University of Enna “Kore”, Enna, Italy; ^5^Recovery and Functional Rehabilitation Unit, Umberto I Hospital, Enna, Italy

**Keywords:** peripheral neuropathies, adaptive sports, Judo training, balance improvement, fall prevention

## Abstract

**Introduction:**

Peripheral neuropathies are progressive conditions characterized by muscle weakness, impaired balance, and reduced quality of life. Rehabilitation programs and adaptive sports have shown promise in mitigating these effects. This paper explores the potential of adaptive Judo to improve motor skills, balance, and overall quality of life in patients with neuropathy.

**Methods:**

We review existing literature, analyze the benefits of Judo’s physical and cognitive demands, and propose adaptive guidelines for its implementation.

**Results:**

This study highlights Judo’s potential as a cost-effective and scalable intervention to support neuropathic patients.

**Conclusion:**

This review emphasizes the evidence-based benefits of adapted Judo training has the potential to transform both the physical and emotional health of neuropathic individuals.

## 1 Introduction

Peripheral neuropathies encompass a diverse group of disorders resulting from damage to the peripheral nerves. These conditions may have a genetic origin, as seen in inherited neuropathies such as Charcot-Marie-Tooth (CMT) disease, or may be acquired, including dysimmune neuropathies like Chronic Inflammatory Demyelinating Neuropathy and related forms. CMT represents a genetically and phenotypically heterogeneous group of progressive inherited disorders, characterized by either nerve demyelination or axonal degeneration (Hoyle et al., 2015; Klein et al., 2013). This condition has a prevalence of approximately 1 in 2,500 individuals (Bird, 2023; Timmerman et al., 2014).

The symptoms of peripheral neuropathies are highly variable and typically include: (i) impaired tactile and proprioceptive sensitivity (Szigeti and Lupski, 2009) that progresses from distal to proximal regions; (ii) muscle weakness (Burns et al., 2010) predominantly affecting the distal portions of the lower limbs, with potential involvement of the upper limbs; and (iii) gait disturbances, resulting in an elevated risk of trips and falls (Li et al., 2019). Individuals with these conditions are significantly more likely to experience falls during daily activities compared to healthy individuals (Li et al., 2019).

These disorders often lead to progressive muscle weakness (Burns et al., 2010) and atrophy in the distal limbs, accompanied by foot deformities such as cavovarus, high arches, flat feet, hammer toes, or foot drop. Additional manifestations include chronic pain, muscle cramps, numbness, paresthesia, and deficits in balance, vision, and hearing, all of which contribute to increasing challenges in performing everyday tasks (Szigeti and Lupski, 2009).

Symptom onset varies widely, typically emerging between the ages of 5 and 15, with significant negative impacts on physical functioning and quality of life (Burns et al., 2010). Evidence from an 8-month adapted motor activity program conducted with a 16-year-old male, comprising two 1-h sessions per week, suggests that balance training may effectively counteract balance loss in patients with CMT1A. Improvements in left plantar flexion strength observed during the program were attributed to enhanced nerve recruitment and muscle fiber hypertrophy in the targeted muscles (Bottoni et al., 2024).

Currently, there is no cure or definitive treatment for CMT disease, and its progression cannot be halted or reversed. While immunotherapies are available for certain acquired neuropathies, they often fail to fully restore nerve function following injury.

Rehabilitation has proven effective in slowing disease progression and preserving functional improvements, but it is frequently costly due to the need for individualized treatment plans, which are not universally covered by national healthcare systems. Conversely, more accessible and cost-effective options, such as stretching, strengthening exercises, and moderate physical activity, can be performed in various settings and offer viable alternatives for managing symptoms.

Telecoaching (TC) has emerged as a promising training model for individuals with CMT disease, as described by Leale et al. (2024). A systematic review of TC-based interventions revealed notable improvements in strength, cardiovascular fitness, functional abilities, gait, and fatigue management. Among the studies analyzed, five focused on resistance training protocols for CMT patients, while two evaluated the effectiveness of interval training. Two studies included only male participants, whereas the remaining five included both genders.

Results indicated that following resistance training, women achieved 80% of normal strength in 8 out of 10 assessments, compared to just 1 for men. Functional improvements in daily activities were observed in both genders, with no significant disparities. Furthermore, another investigation into intensive rehabilitation, conducted over 3 weeks (5 days per week, with sessions ranging from 2 to 4 h per day depending on patient fatigue), demonstrated substantial benefits. Specifically, 32.4% of patients exhibited strengthened proximal lower limb muscles, and 43.2% showed increased strength in distal lower limb muscles (Ferraro et al., 2024).

Neuropathies are frequently associated with reduced quality of life, with numerous studies highlighting a strong correlation between these conditions and depression (Bellofatto et al., 2023; Querol et al., 2021). Engaging in sports has been identified as an effective strategy to enhance overall wellbeing and quality of life while simultaneously reducing social barriers and discrimination faced by individuals with disabilities (DePauw, 2009). Recent research underscores the positive impact of physical activity on individuals with CMT disease. Specifically, CMT patients who participated in sports activities reported better physical quality of life and lower levels of neuropathic pain compared to those who did not engage in such activities (Pazzaglia et al., 2022; Ferraro et al., 2024). Furthermore, a three-week intensive rehabilitation program focusing on improving functioning and balance in patients with mild to moderate CMT demonstrated that regular exercise is crucial to prevent functional decline. This evidence supports the necessity of ongoing exercise programs for maintaining physical capabilities and mitigating disease progression (Ferraro et al., 2024).

Sport-specific multimodal exercise training all-embracing physical and mental exercises, including self-defense techniques could be achieved practicing Judo (International Judo Federation), one of the most widely practiced Olympic Sports Worldwide.

In particular, strong evidence that the systematic practice of Judo can lead to the improvement of functional fitness and psychosocial capacity have been reported by recent review on risks and benefits of Judo training for middle-aged and older people (Palumbo et al., 2023). Those Judo benefits have a positive impact on the quality of life in the adult and elderly population.

Although there is no evidence whether this sport can be adapted to neuropathic subjects, since the benefits of Judo are well suited to the pathological characteristics of this group of diseases.

This communication aims to fill the knowledge-gap first by explaining what Judo is, second on zooming the impact of this discipline practice on balance and fall event prevention and control, third analyzing Judo intervention guideline for fragile people, as subjects suffering from neuropathies. Ultimately, the purpose of this paper is to sensitize and promote further research to improve neuropathic subjects’ quality of life.

## 2 Methods

### 2.1 What is Judo

Judo is a grappling-based combat sport and a modern Japanese martial art that has been included in the Olympic Games since 1964 for men and 1988 for women, establishing itself as a globally recognized sport (International Judo Federation, n.d.). The term “Judo,” derived from Japanese, translates to “the gentle way.” Founded in 1882 by Professor Jigoro Kano, Judo was inspired by the traditional martial art of Jujutsu.

Professor Kano, who as a young man had a frail physique and experienced bullying, began practicing Jujutsu to improve his physical strength and resilience (International Judo Federation, n.d.; Kodokan Judo Institute, n.d.). A distinguished educator and philosopher, Kano redefined Jujutsu’s principle of “defeating strength through flexibility” into Judo’s guiding principles: “maximum efficient use of physical and mental energy” and “mutual welfare and benefit.” In doing so, he eliminated dangerous Jujutsu techniques and prioritized the development of safe falling methods (Kodokan Judo Institute, n.d.).

Judo thus emerged as a comprehensive theoretical and technical system, designed to address the needs of modern individuals. Initially regarded as a self-defense practice, Judo has since gained recognition for its significant educational value, fostering physical fitness, mental fortitude, and character development (Kodokan Judo Institute, n.d.).

### 2.2 Literature review methodology

The literature review was conducted using databases such as PubMed, Scopus, and Web of Science. Search terms included ‘adaptive sports,’ ‘Judo,’ ‘peripheral neuropathy,’ ‘balance improvement,’ and ‘fall prevention.’ Both peer-reviewed and gray literature were considered. Articles were filtered by publication date (2010–2024) and language (English).

## 3 Results

### 3.1 Judo’s impact on balance, fall prevention, and control

**Figure 1:**
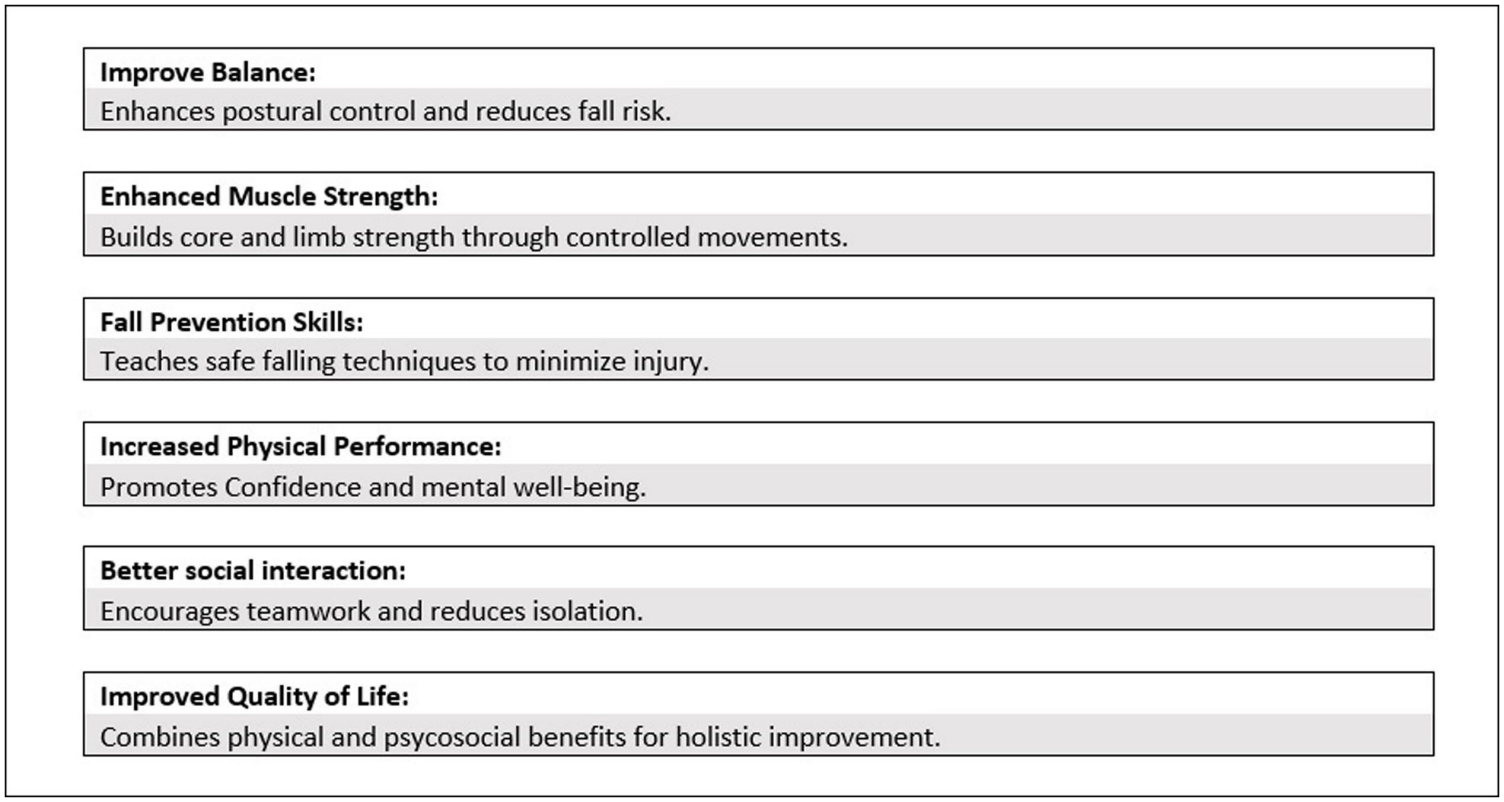
Key benefit of Judo for neuropathic individuals.

Sport-exercise interventions that modulate both physical fitness and cognitive functions have become a promising tool to support subjects with neuropathy (Figure 1).

In this communication, we suppose that Judo’s adaptive training program can improve brain and muscle function in neuropathic subjects.

Because neuropathies cause a decline in the nerves that lead to progressive muscle weakness (Burns et al., 2010) and wasting, reduced muscle strength, and limited range of motion, martial arts can slow the progression of the disease or maintain the acquired level of functioning, of aging population. Judo is a great example of physical activity that could help keep fit and healthy in numerous way (Palumbo et al., 2023).

Postural stability plays a key role in neuropathic subjects’ everyday situations significantly affecting both safety and quality of life.

In contact sports, such as Judo, balance stability maintenance requires the most efficient regulatory and control processes, thus making this discipline a viable tool for improving balance indicators (Palumbo et al., 2023).

Judo sessions include coordination and balance exercises performed barefoot, as well as active engagement in activities and experiences that help people develop and maintain their agility (e.g., displacements in all directions, go to the ground safely and get up), strength (e.g., push and pull exercises with partner, with practitioners moving and often lift the partner), tonicity and flexibility (e.g., ground exercises with stretching and core holding positions), which could help counteract the main degenerative processes of aging (e.g., sarcopenia, osteoporosis) (Palumbo et al., 2023). Judo, in which many basic exercises and movements are based on obtaining balance, could be a strong stimulus to shape this ability. In addition, the correct Judo throw execution requires the disturbance of the partner’s balance (*kuzushi*), which can lead to his/her overthrow.

The fact that Judo training can start during the developmental period bring even greater benefits, overlapping with natural development processes. Given that the onset of some genetic forms of neuropathy can occur in childhood and adolescence, Judo practice could be crucial during the developmental period, because it could bring even greater benefits, counteracting the progression of the disease’s symptoms.

For this reason, Judo practice could be adopted to maintain, even to restore, balance in neuropathic subjects.

Neuropathic subjects are at great risk of fall, due to balance disorders, and related consequences such as injuries and fractures. If we combine this pathology with advancing age, and the associated decreased of physical activity, self-esteem, confidence, strength and balance, the fear of falling increasing heavily, consequently limiting the person’s quality of life.

Since in this situation the event falls dangerously expose to the risk of fractures, with all the possible negative consequences determined by this incident.

The Judo activity, thanks to the learning and the training process of the falling techniques, in an effective learning progression, reducing the fear of falling. This fall control skill supports fragile individuals in both social activities (e.g., walking confidently in crowded places) and more difficult daily activities (e.g., walking on slippery surfaces) (Palumbo et al., 2023). In older judokas, lower levels of concern regarding falling have been associated with higher physical performance in the lower extremities, including strength, balance, and walking ability (Palumbo et al., 2023).

### 3.2 Judo other benefits

An important concern to address is that individuals with neuropathy often report difficulties with vision, which can render traditional training methods less effective. However, the review by Palumbo et al. (2023) highlights that older judokas demonstrate superior dynamic visual acuity and peripheral vision compared to their sedentary peers. In situational sports and martial arts like Judo, which demand rapid adaptations to unpredictable contexts, practitioners develop specific skills and heightened peripheral vision to anticipate or respond to varying challenges (Palumbo et al., 2023).

Judo practice also fosters significant benefits in social interactions and self-esteem across diverse populations. It instills a collaborative ethos, as training inherently depends on mutual learning and cooperation within the group dynamic, which are foundational to partner relationships (Palumbo et al., 2023).

## 4 Discussion

### 4.1 Recommended Judo training guidelines for individuals with fragility

The review by Palumbo et al. (2023) offers a detailed analysis of best practices for teaching and training Judo to individuals with fragility, emphasizing strategies to prevent injuries and optimize benefits. A well-designed Judo program for this population should aim to enhance physical, mental, and social wellbeing, improve quality of life, promote active aging, and reduce the risks of falls, injuries, and disabilities. Safety is a central principle, with training tailored to individual capabilities, regular progress monitoring, and assessments of participants’ functional fitness, athletic abilities, and enjoyment levels.

The methodology should prioritize movement control and precision over speed and power to ensure safety. Exercises should include general warm-ups, basic techniques, prearranged forms such as kata, and specific Judo breakfall techniques (ukemi-waza). Techniques performed from standing positions (tachi-waza) and on the ground (ne-waza) are recommended, while higher-risk techniques like joint manipulation (kansetsu-waza) and strangulation (shime-waza) should be excluded or practiced only within the controlled framework of kata.

Potential barriers to participation, such as fear of falling, fear of contact, shyness, and embarrassment, must be addressed to ensure commitment and effective results. Additionally, considerations should be made for the physical limitations associated with fragility, such as reduced strength, endurance, flexibility, and conditions like osteoporosis and osteopenia. The psychological impact of loneliness, which participants may experience, also warrants attention.

**Figure 2:**
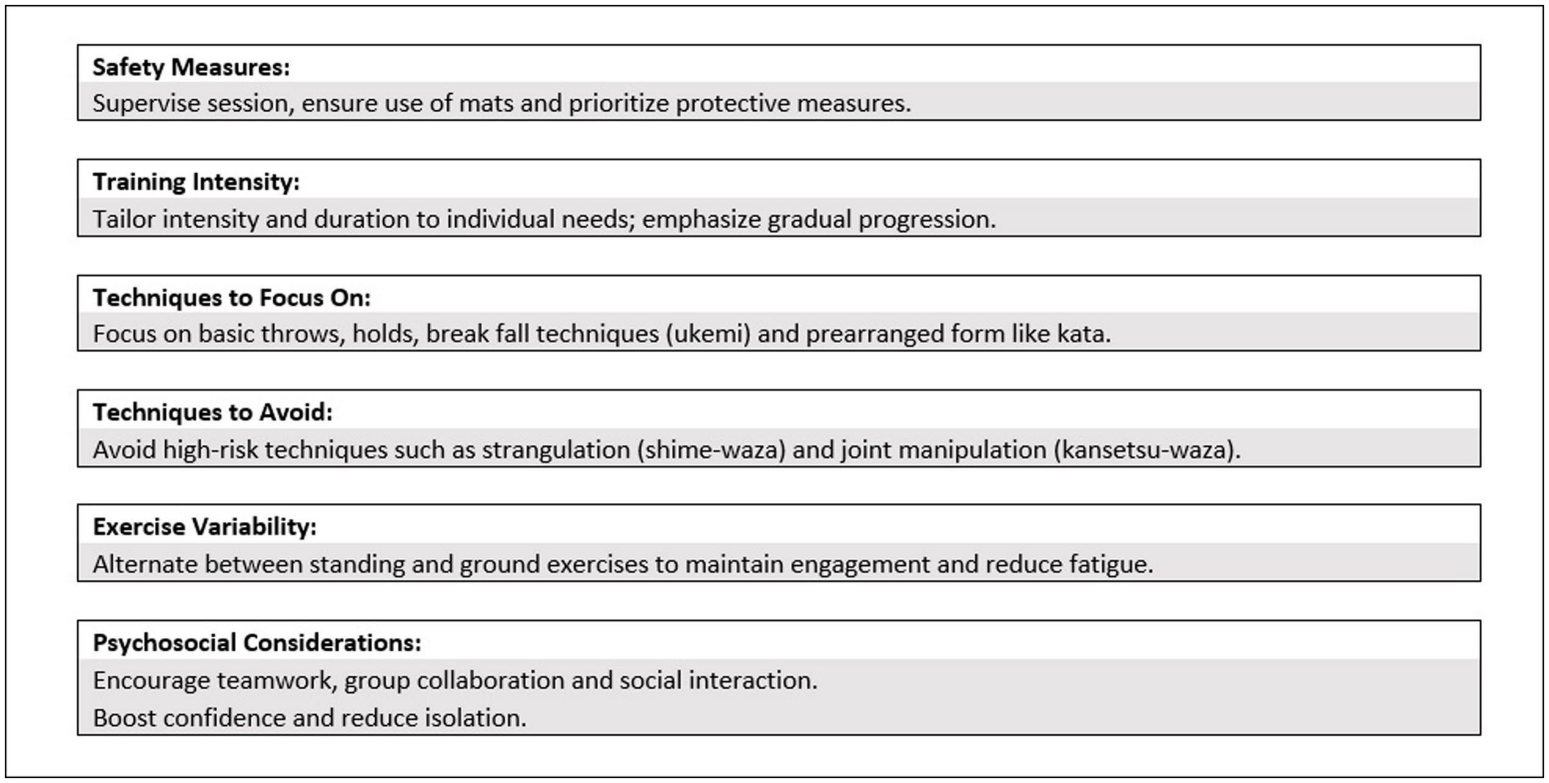
Recommended Judo training guidelines for individuals with fragility.

To prevent fatigue during Judo lessons for individuals with peripheral neuropathy, Prada et al. (2018) recommend varying the exercises between standing and ground activities to balance intensity and engagement. An appropriate work-to-recovery ratio should be maintained, and monotony in learning situations should be avoided to sustain motivation and enhance training effectiveness. These guidelines collectively aim to provide a safe, inclusive, and effective Judo training experience for individuals with fragility (Figure 2).

### 4.2 Strengths and limitations

This mini review highlights the unique potential of Judo for neuropathy management and identifies gaps in current literature. Strengths include its focus on a novel rehabilitation approach and the integration of findings from diverse sources. Limitations include reliance on existing literature without new experimental data and potential bias due to language restrictions.

## 5 Conclusion

### 5.1 Bridging scientific literature and practical application

This review seeks to highlight the crucial role of adaptive sports and martial arts, particularly Judo, as effective interventions for individuals with peripheral neuropathies. Judo, with its structured techniques and emphasis on balance, movement precision, and controlled physical interaction, demonstrates a significant potential to enhance balance, physical performance, muscle strength, and psychosocial wellbeing in this population. The multifaceted nature of Judo training offers not only physical improvements but also contributes to increased self-esteem, social interaction, and overall quality of life.

While the relationship between Judo training and somatosensory feedback in postural control remains underexplored, existing evidence strongly supports the positive impact of Judo on fall prevention in individuals with neuropathies. Through its emphasis on proprioception and dynamic stability, Judo fosters better body awareness and control, key factors in minimizing fall risks. Furthermore, the practice of breakfall techniques (ukemi) is particularly beneficial, as it equips individuals with the skills to fall safely, significantly reducing the likelihood of injury during accidental falls. This aspect alone has profound implications for enhancing quality of life and reducing the fear of falling, a common barrier to physical activity in neuropathic populations.

Moreover, adapted Judo training prioritizes safety and inclusivity, making it a feasible intervention even for individuals with significant physical limitations. By tailoring training intensity and techniques to the individual’s capabilities, adapted Judo mitigates risks while ensuring progressive improvement in functional fitness and postural control. The integration of prearranged forms, such as kata, allows for the development of technical skills without unnecessary strain, while the avoidance of high-risk techniques like strangulation (shime-waza) ensures safety for participants.

In conclusion, our review underscores the evidence-based benefits of adapted Judo training as a safe, practical, and effective intervention for individuals with peripheral neuropathies. By addressing key aspects such as fall prevention, muscle strength, and psychosocial wellbeing, adapted Judo has the potential to transform both the physical and emotional health of neuropathic individuals. As such, it should be considered a valuable addition to therapeutic and rehabilitative programs aimed at improving quality of life and fostering active participation in daily activities.
